# Transcriptomics View over the Germination Landscape in Biofortified Rice

**DOI:** 10.3390/genes12122013

**Published:** 2021-12-18

**Authors:** Conrado Jr. Dueñas, Inez Slamet-Loedin, Anca Macovei

**Affiliations:** 1 Department of Biology and Biotechnology, University of Pavia, Via Ferrata 1, 27100 Pavia, Italy; conradojr.duenas01@universitadipavia.it; 2 Trait and Genomic Engineering Cluster, Strategic Innovation Platform, International Rice Research Institute, DAPO Box 7777, Metro Manila 1277, Philippines; I.Slamet-Loedin@irri.org

**Keywords:** biofortification, germination, *Oryza sativa*, micronutrient deficiency, seed quality, transcriptomics

## Abstract

Hidden hunger, or micronutrient deficiency, is a worldwide problem. Several approaches are employed to alleviate its effects (e.g., promoting diet diversity, use of dietary supplements, chemical fortification of processed food), and among these, biofortification is considered as one of the most cost-effective and highly sustainable. Rice is one of the best targets for biofortification since it is a staple food for almost half of the world’s population as a high-energy source but with low nutritional value. Multiple biofortified rice lines have been produced during the past decades, while few studies also reported modifications in germination behavior (in terms of enhanced or decreased germination percentage or speed). It is important to underline that rapid, uniform germination, and seedling establishment are essential prerequisites for crop productivity. Combining the two traits, biofortified, highly-nutritious seeds with improved germination behavior can be envisaged as a highly-desired target for rice breeding. To this purpose, information gathered from transcriptomics studies can reveal useful insights to unveil the molecular players governing both traits. The present review aims to provide an overview of transcriptomics studies applied at the crossroad between biofortification and seed germination, pointing out potential candidates for trait pyramiding.

## 1. Introduction

Micronutrient deficiency (MD), well-correlated with the global hunger challenge, is plaguing the world population, especially people living in developing countries. At least two billion people worldwide are suffering from micronutrient deficiency due to poverty, health restrictions, cultural or religious practices, and political unrest, and children are the most hit category. In view of the Agenda 2030 Sustainable Development Goals (SDG), MD can be addressed within the SDG 2—“Zero hunger”, which promulgates to end hunger, improve nutrition, achieve food security, and promote sustainable agriculture [1]. In spite of these efforts, it is still predicted that 37 countries will fail to reach this target or even the low hunger status by 2030. Based on the 2020 statistics, 40 countries are currently facing serious levels of Global Hunger Index (GHI), and 11 countries are in alarming levels of GHI, most located in developing countries in Sub-Saharan Africa and Asia [2]. 

Since the 1980s, the prevalence and rampant negative effects of MD have been aggregated under the coined term of “hidden hunger” [3]. Hidden hunger can be considered alarmingly dangerous especially since it describes a state of deficiency that largely occurs without any direct signs and symptoms of the deficiency [4]. This further resonates with the common consumption of a high-calorie diet engrained to cultures that have a high dependency on cereals such as rice, wheat, and maize. For instance, in the Philippines, it was reported that 93.39% of households choose rice as a staple commodity, and the annual average consumption per person is 109.87 kg [5]. Moreover, it has been projected that rice consumption will further increase by 14.6 million metric tons [6]. This is only illustrating a fraction of the total 74% cereal consumption in developing countries based on global consumption of 1250 million tons [7]. The consumed rice grains used by almost half of the world’s population are rich in carbohydrates but low in micronutrients [7,8]. This low nutrition value is partly due to post-harvest processing (e.g., milling, polishing—which removes the aleurone layer containing about 61% minerals) of the grains and the presence of anti-nutrients (e.g., phytic acid) [9]. Rice is culturally eaten as white kernels, and polishing or milling helps increase shelf life by removing oil rice bran that would affect its palatability and marketability if not stored in vacuum containers [10,11].

To alleviate the status of hidden hunger, different approaches (e.g., promoting diet diversity, use of dietary supplements, chemical fortification of processed food) have been made as part of national and international programs. However, such programs can be difficult, especially for people in developing countries where poverty and ill-health are highly spread [12]. Moreover, these approaches alone cannot correct micronutrient malnutrition deficiencies when the populations have limited access to fortified food due to poverty or location [13]. Thus, in the long run, it would be more beneficial to utilize biofortification as a complementary approach for the pre-existing methodologies to effectively alleviate MD. Biofortification is the process of increasing the nutritional level of food staples, such as rice, by modifying the genotypic make-up through conventional breeding, transgenic approach, and genome editing. This approach is considered as one of the most cost-effective and highly sustainable compared with previous approaches because it provides a substantial baseline of bioavailable micronutrients in staple crops [14,15]. Additionally, once produced, it does not require specialized infrastructures and constant monitoring. Rice is one of the best vehicles for biofortification since it contributes from 35 to 59% of the caloric intake of energy for 2700 million people in Asia alone, being the third most cultivated crop in the world [16,17]. 

A crucial trait related to high crop productivity refers to optimal germination and seedling establishment. In this context, seed vigor is defined by ISTA [18] as “the sum of those properties that determine the germination activity and performance of seed lots in a wide range of environments”. Rapid and uniform germination, along with the accumulation of biomass during the initial phase of seedling establishment, are the prerequisites of a successful production line. The germination process is generally defined as a triphasic action. The first phase of germination is characterized by rapid water uptake, thus an increase in seed weight which, in the case of rice, corresponds to the first 20 h imbibition. This is subsequently followed by phase II, or the lag phase, spanning from 20 to 48 h of imbibition, and phase III (after 48 h of imbibition) when the radicle starts to emerge [19]. Different and complex physiological and biochemical events are involved in seed germination, during which embryo cells transit from a quiescent state to a metabolically active state. The early seed germination phases, also called pregermination, contribute to restoring the metabolic activity of the seed and is characterized by dynamic changes, including carbohydrate metabolism, signal transduction, DNA synthesis, gene expression, regulation of redox homeostasis, and DNA repair [20]. High rice agricultural production requires seed stocks with high rates of synchronized germination and low dormancy. Therefore, seed dormancy, a quantitative trait directly related to the activation of the pregerminative metabolism, is among the most studied seed trait in model species and crops, including rice [21,22,23]. Physiologically, seed dormancy could be defined as a temporary failure to complete germination under favorable conditions, an event which is controlled by complex molecular networks [24]. Considering the triphasic germination process, nondormant rice seeds germinate rapidly when they are imbibed at 30 °C [25]. The pregerminative phase includes large modifications in transcript abundances and activation of transcriptional regulatory programs happening between 3 and 12 h of imbibition [26]. It appears that de novo transcription is not strictly mandatory for the early stages of germination, but rather necessary for the subsequent regulation of the germination rate and seedling establishment [27,28]. Hence, in the case of nondormant seeds, the second phase, although it does not include visible morphological changes, is characterized by germination-specific changes that anticipate radicle protrusion and seedling growth. 

High-quality seeds, with efficient and uniform germination, have the potential to improve field emergence, can lead to better suppression of weed growth, and produce high yield even under different conditions [29,30]. With this in mind, mining for key genes controlling seed germination behavior along with the understanding of their molecular mechanisms represents important objectives in rice breeding programs.

Combining the two traits, biofortified, highly-nutritious seeds with improved germination behavior can be envisaged as a highly-desired target for rice breeding. Although currently there are still only a handful of studies looking into this particular aspect, the information gathered from transcriptomics analyses can provide useful insights into the molecular players governing both traits, along with the identification of potential biomarkers to test seed quality. Several studies reported poor germination in different lines of biofortified rice [31,32,33], while in other cases, agricultural biofortification (e.g., soil or foliar application of zinc or selenium) proved to be beneficial for germination [34,35]. The current advances in omics can now provide tools and techniques for the efficient exploration of genetic resources along with a better understanding of the molecular mechanisms involved in trait development. Knowledge about genes or loci governing different traits has proved to be effective in accelerating breeding programs. In addition to this, advances in omics branches like transcriptomics are regarded as efficient tools to further explore rice improvement programs. The present review aims to provide the state of the art relative to transcriptomics studies applied at the crossroad between biofortification and seed germination, pointing out potential candidates for trait pyramiding. 

## 2. Linking Rice Biofortification with Seed Germination

When considering rice biofortification, it is important to keep in mind how the different micronutrients are distributed, including the changes in distribution during seed germination. Using synchrotron X-ray microfluorescence, a study was performed to measure the in vivo mineral distribution patterns and shifts during germination [36]. An element-specific distribution was evidenced in the rice grain, while during germination, iron (Fe), zinc (Zn), and manganese (Mn) accumulated more in the elongating shoot tissues, and calcium (Ca) and potassium (K) were remobilized to the radicle. As essential plant micronutrients, K and Ca are required for proper plant growth and development from the early stages of seedling development. Their activities are important to maintain turgor pressure, plant cell signaling, signal transduction, metabolic reactivation, and meristematic tissue activity [37,38]. On the other side, Fe and Zn are being used during germination for protein synthesis, gene expression, respiration, and oxidative stress tolerance [39,40,41]. 

Still concerning the germinative behavior, different lines of biofortified rice have shown poor germination rates [31,32,33]. One such example relates to the content in phytic acid, a chelating agent that binds Ca, Fe, and Zn, making them less bioavailable during digestion. Low phytic acid (LPA) rice mutants were developed to overcome this problem, but these lines presented different agronomic penalties associated with low grain yield [31,42]. Different mutations in genes involved in the biosynthesis of PA were shown to hinder plant growth and reduce seed viability [32,43,44]. Still, other studies showed that different types of breeding (mutagenesis, hybridization, backcross, and marker-assisted breeding) and selection programs can be employed to improve the germination traits in LPA mutants [45]. Aside from phytic acid, high levels of ascorbate have also been associated with low germination in rice [46]. In cereals, ascorbate is present in high amounts during seed maturation til the begining of dehydration, while it starts to progressively decline afterward since it becomes oxidized to dehydro-ascorbate [47,48]. On the other side, from the point of view of biofortification, for humans, ascorbate is an essential micronutrient that is also able to promote Fe absorption in the gut. Recently, rice lines engineered to constitutively overexpress GDP-L-galactose phosphorylase (*OsGGP*), presented an 8.7-fold increase in ascorbate concentrations [33], although with a considerable decline in germination. The GGP enzyme is responsible for catalyzing the conversion of GDP-L-galactose to L-galactose1-P, representing the first step toward ascorbate biosynthesis. Although this approach was successful in enhancing the levels of ascorbate also in rice grains, it does not investigate why germination is affected.

Differently, other types of agricultural fortification methods, like soil supplementation or foliar spraying with micronutrients, appeared to have positive effects on germination [34,35,49]. An extensive report on the effect of Zn-biofortified rice seeds on grain yield was conducted under field conditions in six countries [34]. This study, supported by the HarvestPlus program, tested the effects (in terms of seedling density, grain yield, and grain Zn concentration) of low-Zn and high-Zn seeds, the latter being the product of biofortification with foliar Zn fertilization in maternal plants grown under field conditions. The results of multilocation field studies over a two-year time course evidenced that seeds biofortified with Zn showed enhanced crop productivity with beneficial effects on seed germination, seedling vigor, and overall crop establishment [34]. In another study, the impact of Zn, selenium (Se), and the simultaneous application of the two micronutrients, were investigated in terms of seedling vigor in biofortified rice [35]. Zn and Se were added to the soil as solution of sodium selenite and zinc sulfate fertilizers at different concentrations and combinations (0, Zn5, Zn10, Zn15, Se1, Zn5 + Se1, Zn10 + Se1, and Zn15 + Se1). The reported results evidenced that single Zn and combined Se-Zn application had a positive impact on both germination percentage and seedling growth. On the other hand, a different study on Se biofortification in rice reported that high concentrations of Se delivered as selenate (SeVI 450 mg L^−1^) completely inhibited germination [49]. Hence, when considering the so-called agricultural biofortification methods, it is always important to keep in mind the dosage effects along with the types of chemicals (e.g., salts) which are being delivered. 

## 3. Transcriptomics Blueprint of Biofortified Rice 

Although transcriptomics data are rich in rice, much fewer studies were dedicated to investigating high-throughput transcript changes concerning biofortification. Rice RNA-seq and microarray data are deposited in specific databases, such as NCBI SRA (https://www.ncbi.nlm.nih.gov/sra), RiceXPro (https://ricexpro.dna.affrc.go.jp/), BAR (http://bar.utoronto.ca/efprice/cgi-bin/efpWeb.cgi), or GEO (https://www.ncbi.nlm.nih.gov/geo/). Whereas the majority of transcriptome profiling has been conducted for stress response, fewer efforts have been dedicated to rice nutritional quality [50]. For instance, in relation to mineral nutrient homeostasis, a study investigating the role of alternative splicing was conducted through whole-transcriptome RNA sequencing on rice roots grown in the presence/absence of different nutrients, indicating that alternative splicing is highly nutrient-specific [51]. The antagonistic interactions between Fe and phosphorus (P) were investigated in rice seedlings through microarray analyses [52]. The study showed that the presence of P affects both Fe availability as well as the regulation of Fe-responsive genes.

In strict relation to biofortification programs, most studies so far focused on the identification of QTLs to pinpoint genetic regions underlying nutritional quality traits [53,54,55,56]. In a recent study, a meta-analysis approach was undertaken to identify QTL hotspots associated with Fe and Zn [57]. The gathered results evidenced that approximately 50% of the genes identified have already been functionally characterized as being directly involved in Fe and Zn homeostasis, while other 37 novel genes were suggested to be correlated with these traits. These studies are important because before selecting suitable genes for the biofortification program, an in-depth functional characterization of expression patterns during micronutrient uptake, transport, and storage is required. Given this, a recent study investigated the transcriptional changes in rice flag leaves following agronomic biofortification with Zn and Se delivered through foliar spraying [58]. Flag leaves play important roles in grain filling, especially in the biosynthesis and translocation of minerals to the seeds [59]. The study evidenced a relatively low number of differentially expressed genes (DEGs) with significant fold changes in response to the imposed treatments. The reasoning behind this appears to be associated with a limited impact on specific pathways (e.g., mineral absorption, translocation) or post-transcriptional regulation [58]. A relevant upregulated (3.2-fold) DEG (*Os03g0103300*) associated with seed germination encodes a QLTG-3-1 protein targeted for low-temperature germinability. Improvement of cold tolerance during germination is important for rice cultivation in tropical areas where severe yield reduction is noticed under low temperatures [60]. 

A landmark study for transcriptomics analyses in biofortified rice addressed the high-Zn trait by using RNA-seq performed on panicles [61]. The experimental setup of the study included the use of two landraces (Chittimutyalu-CTM, Kala Jeera Joha-KKJ) and one popular improved variety (BPT5204-BPT), with different Zn content in polished rice, which were grown under Zn-sufficient soil conditions. Rice landraces with high-Zn levels in polished grains represent a good source to study this trait, but these show lower yields compared to the improved varieties [62]. Numerically, the study reported 563 common transcripts for the three rice accessions, 708 transcripts present only in BPT, 314 transcripts present only in CTM, and 322 found only in KJJ [61]. The most abundant categories of genes were related to transcription factors and transporter families. The study also evidenced that more transcripts were upregulated in BPT, while downregulated genes were more abundant in the landraces. The authors explain these findings based on the fact that breeding programs usually target traits of agricultural interest, which might have induced accumulation of genes that upregulate with the phenotype. On the other side, the majority of DEGs in landraces were associated with uncharacterized proteins, indicating higher genetic variability for this trait. Some of the pinpointed DEGs include well-known Fe/Zn transporters, like NRAMP5 metal transporter and VIT (vacuolar iron transporter), along with less-studied transporter families such as the proton-coupled peptide transporters (POT), identified exclusively in landraces. Another interesting finding was the upregulation of *Os01g0578000*, encoding a DNA repair protein radA (RadA)-like, in the BPT. This may indicate that the improved variety may cope better with DNA damage. Studies in *Arabidopsis thaliana* also indicated that DNA repair players are essential under Zn deficiency [63]. 

Another study that dealt with transcriptomics studied in biofortified rice relates to rice high-folate lines developed through metabolic engineering [64]. Folate, or vitamin B9, is synthesized de novo only by plants and microorganisms, thus, humans are entirely dependent on diet as a source of folates. Some leafy vegetables, legumes, fruits, and fermented products are rich folate sources, whereas most staple crops, including rice, have poor folate content [65]. Hence, folate deficiency is an important problem, which is being tackled also through folate biofortification using plant breeding or metabolic engineering [66]. For instance, high-folate Nipponbare rice lines were produced by overexpressing *A. thaliana* GTP cyclohydrolase I (*GTPCHI*, G) and ADC synthase (*ADCS*,A) genes, encoding the first enzymes in pterin and p-ABA biosynthesis, both placed under the control of a rice endosperm specific promoter [67]. These lines were then used in transcriptomics profiling by microarray hybridization carried out on developing rice seeds to investigate the effect of folate enhancement on seed metabolism [64]. A relevant observation from this study is the fact that the expression of genes directly involved in folate biosynthesis remained unchanged in the seeds of transgenic lines. Nevertheless, several identified DEGs were linked to folate metabolism, e.g., iron-sulfur clusters, amidophosphoribosyl transferase (Os01g65260), FRA10AC1 (Os11g4200), and C-methyltransferases. For the latter, as folates play important roles in the methylation cycle, the upregulation of methyltransferases can be related to folate enhancement. A few genes involved in the control of seed size (*Os02g13420*—putative leucine-rich repeat receptor protein kinase, *Os12g43640*—putative receptor-like protein kinase) also resulted to have altered expression in the transgenic lines. The high demand for folates in actively dividing tissues [68] may be related to changes in the expression of genes involved in seed development. Similar work has analyzed the transcriptomic landscape of high-tryptophan transgenic rice lines expressing a mutated anthranilate synthase (*OASA1D*) gene [69]. Particularly, these lines accumulated large amounts of free tryptophan (Trp) but showed germination and seedling growth penalties. Although the *OASA1D* lines showed enhanced levels of anthranilate, tryptamine, and serotonin, only very small changes (namely 22 significant DEGs covering Trp- and IAA-related pathways) were observed in the overall plant transcriptome. Albeit the authors did not investigate the reasons behind the poor germination effect, other studies in wheat reported that since tryptophan is a precursor of indoleacetic acid (IAA), both Trp and IAA treatments resulted in inhibited germination; this is explained by the antagonistic effect that IAA has with auxin [70], one of the main promoters of germination. Another study investigating the germination in the *OASA1D* transgenic rice lines reported that IAA levels were enhanced by at least two-fold compared to those in the wild-type [71], in agreement with the inhibitory effect of IAA on seed germination. 

When considering rice starch content, several studies were conducted to improve its digestibility by developing varieties with different proportions of amylose and resistant starch (RS) fractions, mainly because increased amylose or long-chain amylopectin is favored to reduce the glycemic index (GI) [72,73,74]. A transcriptomics analysis performed on high- and low-GI rice varieties investigated the relation between starch metabolism, seed storage, and germination [75]. Interestingly, the study reported that the mobilization patterns of several components (e.g., resistant starch, total starch, free sugars, amylose, and amylopectin chains) during seed germination were analogous to digestion in the human gastrointestinal tract. The differences in transcriptomic profiles were related mainly to starch storage pathways (e.g., granule bound starch synthase, starch synthase, gluteins), cell wall metabolism (e.g., cellulose, xyloglucan, arabinogalactan, expansins), lipid storage pathways (e.g., phosphoethanolamine N-methyltransferase, digalactosyldiacylglycerol synthase), and specific transcription factors (e.g., MYB, GAMYB, bHLH, WRKY75). The observed differences pinpoint mainly to the importance of seed storage pathways in influencing digestibility [75]. However, other studies linked starch metabolism with seed germinability, indicating that enhanced starch accumulation can be related to improved germination [76,77], while repression of SBE (starch branching enzyme) in barley and maize resulted in altered starch component and amylopectin structure which negatively affected both germination and seedling establishment [78]. 

## 4. Transcriptomics Scenario in Relation to Rice Germination 

Seed germination is a complex trait regulated by multiple genes and interactions with environmental factors. As seed germination is essential for successful crop establishment and production, multiple studies looked into the genetic and molecular aspects of this important trait also in rice. Multiple QTLs (e.g., qLTG3-1, Sdr4, AG1, AG2, qSE3, qGR6.2) involved in the control of seed germination have been reported in rice [79,80,81,82,83]. Among these, qLTG3-1 is related to the weakening of seed tissues during germination [84], Sdr4 is associated with seed dormancy [85], AG1/AG2 with germination under submergence [82], while qSE3 and qGR6.3 are associated with germination under salinity conditions [81,83]. Many such studies indicate that germination speed is often associated not only with seed weight, size, and seed dormancy, but also with endosperm weakening, hormones, and storage metabolism. Thus, being such a complex and dynamic stage in plant development, changes in the seed germination transcriptome are also expected. High-throughput analyses suggest that such transcriptome changes may reflect alteration not only in the dormancy status but also in germination vigor and seedling growth [86]. Hence, this section gathers advances made in transcriptomics research concerning rice seed vigor, seed development, dormancy, germination, and priming. 

Regarding seed vigor, a recent article discussed the implications of the *OsIPMS1* (isopropylmalate synthase 1) gene in this process [87]. This gene encodes an enzyme involved in leucine (Leu) biosynthesis [88] and, in rice, it has been shown to play a role in seed dormancy release [89]. This is probably because the ketogenic amino acid Leu can be degraded to acetyl-CoA, a precursor for the biosynthesis of gibberellic acid [90]. By disrupting the *OsIPMS1* gene with the CRISPR/Cas9 system, low seed vigor was observed (in terms of low germination speed and seedling growth), probably also due to the reduction of amino acids in germinating seeds [87]. A transcriptome analysis conducted on these *OsIPMS1* rice mutants identified 1209 DEGs during early germination, most of which were involved in protein processing, glycolysis, or carbohydrate metabolism. The study also evidenced enhanced starch mobilization and changes in amino acid biosynthesis (10–50% lower levels of Leu, Ile, Phe, Tyr, Thr, Cys, Ser, Gly, Val, His, and Pro) during seed germination. Moreover, *OsIPMS1* gene expression was investigated also during seed priming, a well-known technique applied to improve seed germination by conducting controlled imbibition steps followed by dehydration. Increased priming duration brings seeds to exceeding a critical threshold when seeds lose their desiccation tolerance and consequently do not survive dry-back [91,92]. To this point, after testing several priming timepoints (4, 8, 12, 24, 30, and 36 h), the 12 h treatment resulted in a higher germination index and lower T_50_ (time required for 50% of seeds to germinate) in the *OsIPMS1* defective seeds compared to those of unprimed seeds [87]. Simultaneously, qRT-PCR analysis revealed that the mRNA levels of *OsIPMS1* peaked at 4 h and 8 h in primed seeds while subsiding after 12 h of priming treatments. Hence, the study concluded that the *OsIPMS1* gene may be also used as an indicator to identify the best timepoint to stop priming treatments in rice. 

In a different study, when a chloroplastic small heat shock protein (*sHSP26*) from wheat was overexpressed in rice and Arabidopsis plants, Chaudan et al. [93] demonstrated that these transgenic plants had improved seed development and maturation. Microarray data of transgenic Arabidopsis plants indicated that the overall mRNA levels remained unchanged, suggesting that the observed effects were mainly due to sHSP26 overexpression [93]. Still associated with seed development, an extensive RNA-seq analysis was conducted to unveil the molecular mechanisms underlying rice endosperm development [94]. Alongside the fact that the endosperm is the main part of mature seeds with important storage properties, the study by Gao et al. [94] also pinpointed the importance that programmed cell death (PCD) plays during its development. PCD, a process that is dedicated to ensuring the correct occurrence of growth and developmental processes in plants, is characterized by nuclear-cytoplasmic shrinking, dilatation of endoplasmic reticulum, chromatin condensation, formation of apoptotic body, etc. [95,96]. In the case of seeds, endosperm cell death, a particular example of PCD in plants, begins with nucleus degeneration [97] and continues with aleurone cell development upon germination, defined as yet another form of PCD [98]. Therefore, by sampling rice endosperms from three different stages (3, 6, and 10 DAP (day after pollination)), Gao et al. [94] compiled a transcriptomics landscape of endosperm development, pinpointing that many genes related to oxidative phosphorylation, spliceosome, and ribosomes were highly expressed during the early stages, while genes involved in plant defense/stress response and carbohydrate metabolism were representative of later stages. In direct connection with PCD processes, the authors identified several upregulated genes, like *AIP5* (positive regulator of PCD in the tapetum), *BIRH1* (a DEAD-box RNA helicase), *LOL2* (zinc finger encoding protein involved in rice growth), cystatins (cysteine proteinases), along with downregulated genes, among which the majority were involved in ABA metabolism. 

At the crossroads between seed development and dormancy, the study by Huh et al. [99] focused on comparative transcriptomics performed in developing caryopses from two rice cultivars with contrasting dormancy levels. The two rice accessions (Gopum and Samgwang) were chosen to be representative for preharvest sprouting (PHS or vivipary, associated with poor grain quality and yield reduction) and deep dormancy (related to ununiform germination). Transcriptomic profiles, compared at early (3–6 DAH, day after heading), middle (25 DAH), and late (40 DAH) developmental stages reported that the most pronounced changes were identified in caryopses at 25 DAH, a phase when differential dormancy was most notable as well. Because most DEGs (strongly up- or downregulated) between the two cultivars were involved in the seed maturation process, the authors concluded that seed dormancy is highly correlated with the transcriptomic alteration that occurs during this process. In another study, transcriptomic profiling of seed dormancy was assessed in one red rice cultivar, focusing on sampling caryopses before (dormant) and after dry-after-ripening (nondormant) [100]. Among the differences evidenced in this study, it is indicated that in nondormant seeds, glycolysis seems to be preferentially directed to alcoholic fermentation, whereas alanine production is favored in dormant ones. The study also provides an exhaustive description of all the different factors and pathways underlining the impairing effect of dry-after-ripening associated with dormancy breaking, the roles of nitrogen and carbon metabolism, and the involvement of cell wall modifying enzymes, seed storage proteins, transcription factors, and phytohormones. One relevant finding reported in this study relates to the involvement of chromatin modifications in the transition from dormancy to germination. Among the chromatin remodeling key players discussed in this context are histone deacetylases (e.g., HDA19), histone acetyltransferases (HATs), the SWI/SNF chromatin-remodeling ATPases, SYD, (putative helicase), and OsRDR4 (RNA-dependent RNA polymerase). The activity of these factors is closely associated with phytohormone pathways. Namely, it is suggested that the repression of auxin-responsive genes can involve the activity of HDA19, leading to a more compact chromatin state. Differently, the SWI/SNF complex aids to overcome the repressed chromatin state consequent to auxin sensing, and in the presence of SYD, HATs are recruited to revert the compact or repressed chromatin state [100]. 

Aside from the importance of chromatin remodeling factors in seed dormancy and germination, other studies discussed the implications of DNA repair players in these processes. For instance, a recent study reported that suppressing the activity of the *OsPARP1* (poly ADP-ribose polymerase 1) gene in rice resulted in delayed germination [101]. *PARP* genes play important roles in the regulation of DNA damage as well as plant development and immune responses [102]. Other reports, looking into the effects of γ-ray treatments on rice seed germination, reported modulation of specific genes involved in the TC-NER (Transcription Coupled—Nucleotide Excision Repair) repair pathway (e.g., *OsXPB2, OsXPD, OsTFIIS*) and several other helicases [103,104]. Since DNA damage repair proteins usually require changes in the chromatin state to access the damaged sites [105,106], it is quite relevant to understand the influence that these networks can have on maintaining genome integrity during seed germination [107,108]. Concerning germination dynamics, a comparative transcriptome study between rice and barley, evidenced that chromatin structure and remodeling pathways were more active during late germination while cell wall metabolic pathways and peroxidases were most active during early germination [109]. Another transcriptome comparative study focused on investigating indica (YZX) and japonica (02428) varieties bred in China, with different germination behavior; namely, the indica accession was characterized by faster germination speed and seedling growth compared to those of the japonica one [110]. The first line of data assessment focused on the identification of DEGs that were common to both varieties. This approach revealed that mostly amino acids, nucleotide degradation, lipid metabolism, and cell wall metabolism were continuously enriched in the two accessions during the two days of germination monitoring. Subsequently, the authors looked at the DEGs which may have an influence on the diverse germination patterns, pinpointing mainly differences in ROS-scavenging mediated pathways (e.g., ascorbate-glutathione, phenylpropanoid, flavonoid, stilbenoid, diarylheptanoid, gingerol biosynthesis). 

Another layer of transcriptional regulation is associated with the activity of microRNAs (miRNAs), a class of small noncoding RNA that inhibits gene expression by complementary binding to mRNA targets [111,112]. These ubiquitous molecules are involved in plant growth, development, and stress response. To investigate the roles of miRNAs during rice seed germination, He et al. [113] performed deep sequencing analyses on Nipponbare seeds after 12 and 24 h of imbibition with water. Their work resulted in the identification of a total of 289 miRNA loci, out of which 59 were known and 230 were novel rice miRNAs. Some miRNAs were shown to be much abundant in imbibed seeds (e.g., miR319, miR168, miR156, miR166, miR159), whereas a unique miRNA landscape was evidenced in dry seeds, considering that only a few miRNAs were mapped and also enriched (e.g., ptc-miR6478, OsmiR-201) in this category of materials. Hence, the strong turnover of miRNAs profiles from dry to imbibed seeds is indicative of the presence of miRNA-mediated regulatory mechanisms rapidly activated following imbibition. These mechanisms mostly involved the activity of transcription factors (e.g., SPLs, ARFs, TCPs, MYBs) and hormone regulation pathways (e.g., auxin, ABA, GA), since, among the predicted targets of these miRNAs, these are major represented categories. 

Among cereals, a particularity of rice is its ability to carry anaerobic germination in flooded soils. This is possible because seedlings prioritize the elongation of coleoptile over the development of roots, allowing the coleoptile to reach the aerated water surface and then promote oxygenation of developmental tissues [114,115]. Several recent studies have looked into the transcriptomics overview of rice germination under hypoxia, anoxia, and reoxygenation [116,117,118,119,120]. For instance, a microarray transcriptomics profile was carried out in rice (cv. Dongjin) embryos incubated for 24 h under aerobic or anaerobic conditions in the presence/absence of sugar starvation [120]. Considering the impact of sugar (hence, energy requirements of germinating embryos), the study evidenced that the aerobic/anaerobic conditions have different sugar regulation patterns; namely, anoxia induced more downregulation (434 genes) rather than upregulation (96 genes), while the opposite was observed in the case of aerobic conditions (three-fold higher rates of upregulation). In the case of anoxia, the majority of responsive genes were overlapping with low-energy-responsive genes (e.g., α-amylase, hexokinase, ATP dependent phosphofructokinase, pyruvate orthophosphate dikinase, pyruvate decarboxylase, alcohol dehydrogenase). The fact that many of the analyzed genes resulted to be co-responsive under the different conditions implies that the signaling pathways are closely interconnected with each other, and most probably involve ABA [120]. Similar considerations were drawn from another study where transcriptomics of weedy and cultivated rice grown under hypoxia were compared [119]. Additionally, in this case, oxidative phosphorylation was enhanced in weedy rice compared to that in cultivated rice, indicative of more effective energy metabolism during hypoxia. Other than the transcriptional changes during hypoxia/anoxia, another study also investigated the epigenome changes during lack of oxygen as well as during reoxygenation [117]. The study reports striking similarities (more than 80%) in the transcriptome and no differences in the epigenome (in terms of DNA methylation) of 24 h seedlings grown under aerobic and anoxia conditions. An interesting finding relates to the fact that upon reoxygenation, the DNA methylation pattern followed a model similar to what is observed in dry seeds, explained in terms of “resetting the molecular clock” to allow rapid changes and subsequent cell division [117]. Important work was carried out to quantify the interaction of specific QTLs responsible for submergence (SUB1) and anaerobic germination (AG1) [118]. While SUB1 provides tolerance to submergence by dimming the carbohydrate catabolism through the SUB1A-1 ethylene-responsive transcription factor [121], AG1 promotes mobilization of endosperm reserves to enhance coleoptile elongation through the activity of TPP7 (Trehalose-6-phosphate phosphatase 7) [122]. Changes in the transcriptome of four genotypes (IR64, IR64-AG1, IR64-SUB1, IR64-AG1,SUB1) were registered in a period of 2–14 days of complete submergence. The gathered data indicate that while AG1 and SUB1 individually have a similar effect on biomass, soluble sugars, and starch during prolonged submergence, trait pyramiding, however, resulted in epistatic interaction between TPP7 and SUB1A-1, manifested through late elongation growth, low germination, and survival rate. The transcriptomics landscape evidenced both time- and genotype-dependent regulation of genes involved in DNA repair (e.g., replication proteins RPA70B, RPA32, DNA replication factor CDT1, mini-chromosome maintenance MCM2, and MMC7), cell cycle (e.g., cell division control CDC6, cyclin-dependent kinases CDKB2;1 and CYCB2;2), chromatin modification (e.g., DNA methyltransferase 1B, chromomethylase 3A), carbohydrate catabolism (e.g., sucrose non-fermenting-1-related protein kinase SnRK1A, α-amylase, pyruvate decarboxylase PDC1, alcohol dehydrogenase ADH1), and cell elongation (expansins EXPA1, EXPB11, EXPB7) [118]. Additionally, another study conducted comparative transcriptomics between primed and nonprimed seeds germinated under submergence conditions [116]. The authors initially showed that seed priming with selenium (60 μM) and/or salicylic acid (100 mg L^−^^1^) resulted in enhanced germination and seedling growth during submergence. The transcriptomic analysis carried out on four-days-old primed/unprimed seedlings, identified more than 2000 DEGs induced by both priming agents, pinpointing common mechanisms of priming-induced tolerance to submergence. These DEGs were related to carbohydrate metabolism (mainly starch degradation), response to oxidative stress (*GAPDH*, *NADH-GOGAT*, peroxidases), along with several expansins (*EXPA7*, *EXPA16*) linked with coleoptile elongation under submergence, and transcription factors (e.g., *ERF47*, *ERF108*, *ERF35*, *ERF20*, *ERF79*) [116].

## 5. Concluding Remarks 

**Figure 1:**
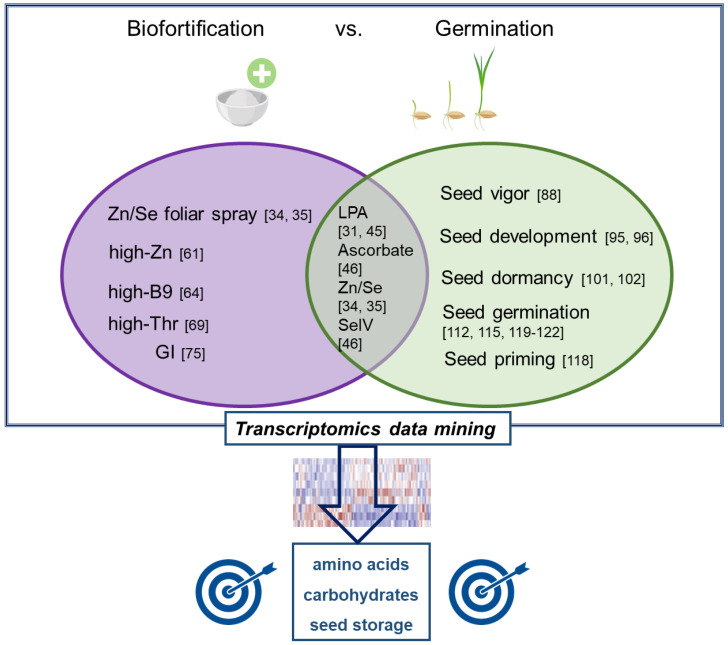
Schematic representation summarizing transcriptomics studies applied at the interface between rice biofortification (left) and seed germination (right). The overlapping region includes studies where germination aspects were investigated in biofortified rice. The blue square represents putative target pathways, mined from transcriptomics studes, that may result in trait pyramiding. References are indicated as numbers.

With the present review, we aimed at providing an overview of transcriptomics investigations carried out so far at the interface between rice biofortification and seed germination. A summary of what is discussed here is provided in Figure 1, evidencing studies related to germination in biofortified rice as well as potential targets to be addressed for putative trait pyramiding. The idea behind pooling together these two important traits is in line with the current needs to improve nutrition (offered by biofortification approaches) and enhance productivity (achievable also through improved germination) to address some of the top SDGs articulated in Agenda 2030. 

The few studies where seed germination was evaluated in biofortified rice lines indicate that most traits (e.g., LPA, high-ascorbate, high-tryptophan) have negative effects on the germination behavior [31,45,46,69], while agronomic approaches with micronutrient supplementation (e.g., Zn, Se) can also have beneficial effects depending on dosage and type of treatment [34,35]. When these positive outcomes on germination were reported, transcriptomics studies evidenced also the upregulation of genes with specific roles in this process, such as QLTG3-1, reported to improve germination under low temperatures [58], part of a QTL with specific functions in weakening seed tissues during germination [84,123]. On the other hand, enhanced tryptophan production in rice seeds led to germination penalties, due to interferences between the IAA and auxin pathways [69]. Differently, when considering starch and GI, approaches dedicated to improving nutritional aspects could also lead to better germination, likely due to changes observed in starch and lipid storage pathways, cell wall metabolism, and the activity of specific transcription factors [75]. 

In rice seeds, the transcriptomic landscape was overseen during various stages of seed development and germination, including dormancy and vigor. Many genotype-, stage-, and treatment-specific factors were identified along with common players, transversal to other species as well [94,100,109,110,117,118,120]. In relation to this transversality, recently, more emphasis is being placed on mechanisms involving the dynamics of chromatin remodeling and DNA damage response (DDR), a pathway gathering together important downstream processes like DNA repair, cell cycle regulation, and PCD [124,125]. During the different phases of seed germination, the expression patterns of numerous genes are changing, and chromatin remodelers are necessary to regulate the gene switch to promote seed germination. DDR is activated already from the early stages of seed imbibition [20,108,125] and correlations with chromatin modifiers were revealed when different inhibitors (trichostatin A, sodium butyrate) were used [107,126,127]. However, no direct links could be drawn so far between chromatin status and rice biofortification. Nevertheless, a transcriptomics study in barley evidenced novel models where different chromatin remodeling factors (e.g., histone methyltransferase Sdg2, histone acetyltransferase Hac1), along with other players like auxin and ethylene signaling, circadian clock, and storage proteins, are involved in maintaining mineral homeostasis [128]. 

Based on the transcriptomics data analyzed in this work, when screening for common players shared between attributes related to physiological and nutritional seed quality in rice, pathways involving carbohydrates, amino acids, and seed storage, seem to be most prevalent. Therefore, improved nutrition and germination of rice seeds could be more effectively addressed in the future, taking into consideration amino acid and carbohydrate composition as well as seed storage proteins. However, considering the shortage of transcriptomics data related to rice biofortification (at least compared to that of other traits), more studies are encouraged to better understand the complexity of the overall regulatory mechanisms behind such different products.
